# miR-21: a non‐specific biomarker of all maladies

**DOI:** 10.1186/s40364-021-00272-1

**Published:** 2021-03-12

**Authors:** Ana E. Jenike, Marc K. Halushka

**Affiliations:** Department of Pathology, Johns Hopkins University School of Medicine, Ross Bldg. Rm 632B, 720 Rutland Avenue, MD 21205 Baltimore, USA

**Keywords:** microRNA, miR-21-5p, Biomarker, Plasma, Serum, cancer, Heart disease

## Abstract

miRNA-21 is among the most abundant and highly conserved microRNAs (miRNAs) recognized. It is expressed in essentially all cells where it performs vital regulatory roles in health and disease. It is also frequently claimed to be a biomarker of diseases such as cancer and heart disease in bodily-fluid based miRNA studies. Here we dissociate its contributions to cellular physiology and pathology from its potential as a biomarker. We show how it has been claimed as a specific predictive or prognostic biomarker by at least 29 diseases. Thus, it has no specificity to any one disease. As a result, it should not be considered a viable candidate to be a biomarker, despite its continued evaluation as such. This theme of multiple assignments of a miRNA as a biomarker is shared with other common, ubiquitous miRNAs and should be concerning for them as well.

## Introduction

MicroRNAs (miRNAs) are a class of small regulatory RNA. They have important functions in health, disease, and development [1, 2]. miRNAs have a fairly consistent numerical naming convention and hundreds to thousands of highly-conserved miRNAs are known across most species [3, 4]. Because miRNAs are stable in bodily fluids, they have been considered as potential biomarkers of disease [5, 6]. Scores of papers have assigned a litany of miRNA biomarkers to a plethora of different disease states [7–9]. Amongst miRNAs, miR-21-5p (miR-21) is one of the most highly-expressed and highly-studied.

miR-21 was among the first identified microRNAs and is located within the Vacuole Membrane Protein 1 (VMP1) locus on chromosome 17 [10]. It has been implicated in both neoplastic and non-neoplastic pathologies through many of its gene targets. Three of the main targets of miR-21 are Phosphatase and Tensin Homolog (PTEN), Tropomyosin 1 (TPM1), and Programmed Cell Death 4 (PDCD4) [11–14]. The regulation of miR-21 is more complex than most miRNAs. It is transcribed from both a ~ 3.5 kb and a ~ 4.3 kb pri-miR-21, from within the VMP1 locus. The transcription of miR-21 is regulated by hypoxia and cytokines, such as interferon [15, 16]. Further post-translational regulation occurs through transforming growth factor beta (TGFβ) mediated events [17].

Functionally, miR-21 has been assigned a variety of activities. In both neoplastic and non-neoplastic disease, the down regulation of miR-21 increased the rate of cell death, the exact target of this is unknown, though possibilities are HIF-1α, PTEN, and PDCD4 [16, 18, 19]. miR-21 increases cell migration through TPM1 and PDCD4 in neoplastic disease. The upregulation by cytokines indicates a role in inflammation. In cardiovascular disease, miR-21 increases fibrosis and cardiac hypertrophy [20, 21]. Due to these important functional activities, miR-21 has been targeted for therapeutic intervention in neoplastic and other diseases [22–24]. Outside of its role as a biomarker, we refer the reader to these excellent reviews of miR-21 function [25–27].

## miR-21: a biomarker across a spectrum of diseases

**Fig. 1:**
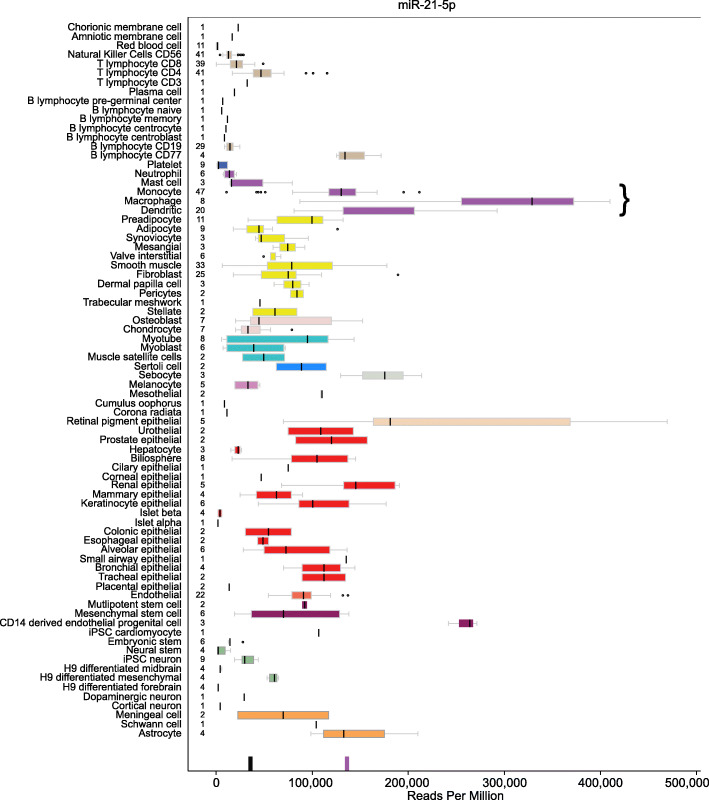
miR-21 expression across 78 cell types. While miR-21 is robustly expressed in most cell types, its highest expression levels appear in macrophages, monocytes, and dendritic cells. Image modified from the UCSC Genome Browser

Not only is miR-21 highly-expressed, it is also ubiquitous across most cell types. However, its common abundant expression is not consistent cell to cell. It has the highest reported levels of expression in macrophages, monocytes and dendritic cells based on cell-specific surveys [28, 29]. From the University of California, Santa Clara (UCSC) Genome Browser Human cellular microRNAome barCharts, the median reads per million (RPM) of miR-21 amongst 75 combined macrophage, monocyte and dendritic cells was 137,021 RPM whereas it was only 38,281 RPM amongst 470 other cells of a combined 75 cell types (Fig. 1). Because of the abundance of miR-21, it is easily measured from bodily fluids such as plasma, serum, urine, peripheral blood mononuclear cells (PBMCs), and other spaces. Across thousands of studies of miRNAs as biomarkers, it frequently appears as having altered expression.

Table 1 lists a subset of studies and diseases in which miR-21 has been implicated as a nonneoplastic biomarker and Table 2 lists cancer biomarker studies. As one can see from the data, miR-21 is implicated as a biomarker in no less than 29 diseases or processes. As far back as 2014, it had already been implicated in 10 non-neoplastic diseases and multiple cancers [30, 31]. Of note, miR-21 is elevated in each of these disease states ranging from cardiovascular disease to cancer. These 45 publications span from 2008 to 2020 and have over 5,000 collective citations (median 68) indicating they are well-known studies and that miR-21 is continued to be pursued as a biomarker.

**Table 1:** Biomarker status of elevated miR-21 across 16 non-neoplastic diseases

Disease Group	Disease	Sample size	Fluid Source	Additional miRNA biomarkers	Predictive or Prognostic	Citation
Cardiovascular Diseases						
1	Myocardial infarction	272	Plasma	miR-1, miR-499, miR-423, miR133a	Predictive	[32]
1	Myocardial infarction	66	Plasma	--	Predictive	[33]
2	Coronary artery disease	52	Plasma	miR-23a, miR-34a	Predictive	[34]
2	Coronary artery disease	147	Plasma	miR-133b	Predictive	[35]
3	Acute coronary syndrome	62	Plasma and PBMC	miR-146, miR-155	Predictive	[36]
4	Chronic cardiovascular disease	60	Peripheral blood	miR-92a, miR-222, miR-130a	Predictive	[37]
5	Left ventricular fibrosis in aortic stenosis	132	Plasma	--	Prognostic	[38]
6	Cardiac arrest	28	Plasma	miR-122	Prognostic	[39]
Non-cardiovascular Diseases						
7	Crohn Disease	78	Serum	miR-16, miR-484, miR-30e, miR-140, miR-192, miR-93, let-7b, miR-195, miR-106a, miR-20a	Predictive	[40]
8	Dengue Fever	6DC / 72VC	Serum	miR-146a	Predictive	[41]
9	Hepatitis C	72	Serum	miR-27a, miR-106, miR-122, miR93, miR-199, miR-23b	Predictive and prognostic	[42]
9	Hepatitis C	110	Serum	--	Prognostic	[43]
10	Multiple sclerosis	48	PBMC	miR-146a, miR-146b	Predictive	[44]
11	Non-alcoholic fatty liver	403	Serum	miR-34a, miR-122, miR-145, miR-451	Predictive	[45]
12	Pneumonia	176	Serum	miR-155, miR-197, miR-182	Predictive	[46]
13	Pulmonary fibrosis	62	Serum EV	--	Prognostic	[47]
13	Pulmonary fibrosis	130	Serum	miR-155	Predictive	[48]
14	Renal fibrosis	42	Serum	--	Predictive	[49]
15	Systemic lupus erythematosus	145	Plasma	miR-126	Predictive	[50]
15	Systemic lupus erythematosus	85	PBMC	miR-155	Predictive	[51]
16	Type 2 diabetes complications	300 DC / 40 VC	Plasma / CAC	miR-126	Prognostic	[52]

Key: *DC* Discovery Cohort; *VC* Validation Cohort; *EV* Extracellular Vesicles; *PBMC* Peripheral Blood Mononuclear Cells; *CAC* Circulating Angiogenic Cells

**Table 2:** Biomarker status of elevated miR-21 across 13 neoplasias

Disease Group	Disease	Sample size	Fluid Source	Additional miRNA biomarkers	Predictive or Prognostic	Citation
1	AIDS-related non-Hodgkin lymphoma	125	Serum	miR-122, miR-223	Predictive	[53]
2	B-cell lymphoma	157	Serum	--	Prognostic	[54]
2	B-cell lymphoma	103	Serum	miR-155, miR-210	Predictive	[55]
3	Breast cancer	122	Serum	--	Predictive/prognostic	[56]
3	Breast cancer	120	Serum	miR-92a	Predictive	[57]
4	Colorectal cancer	330	Serum	miR-92a	Prognostic	[58]
4	Colorectal cancer	60DC / 40VC	Plasma	miR-1, miR-133a, miR-31, miR-135b	Predictive	[59]
4	Colorectal cancer	24DC / 342VC	Serum	--	Predictive/prognostic	[60]
5	Esophageal cancer	42	Serum	--	Predictive	[61]
6	Gastric cancer	42	Plasma	--	Prognostic	[62]
6	Gastric cancer	20DC / 120VC	Plasma	miR-223, miR-218	Predictive	[63]
7	Glioma	112	Plasma	miR-15b	Predictive	[64]
7	Glioma	60	Plasma	miR-128, miR-342	Predictive/prognostic	[65]
8	Hepatocellular carcinoma	80DC / 453VC	Serum	--	Predictive/prognostic	[66]
8	Hepatocellular carcinoma	57	Serum EV	miR-144	Predictive	[67]
8	Hepatitis B/hepatocellular carcinoma	137DC / 407DC / 390VC	Plasma	miR-122, miR-192, miR-223, miR-27a, miR-26a, miR-801	Predictive	[68]
9	Hodgkin lymphoma	62	Plasma	miR-494, miR-1973	Predictive	[69]
10	Lung cancer	176	Serum	miR-155, miR-197, miR-182	Predictive/prognostic	[46]
11	Osteosarcoma	40DC / 48VC	Plasma	miR-221, miR-106a	Prognostic	[70]
11	Osteosarcoma	95	Serum	--	Prognostic	[71]
12	Pancreatic cancer	50DC / 176VC / 68DC / 137VC	Serum	miR-20a, miR-24, miR-25, miR-99a, miR-185, miR-191	Predictive/prognostic	[72]
12	Pancreatic cancer	74	Plasma	miR-483	Predictive	[73]
13	Prostate cancer	28	Serum	miR-106b, miR-144, miR-375	Predictive	[74]
13	Prostate cancer	71	Plasma	miR-141, miR-221	Predictive	[75]

Key: *DC* Discovery Cohort; *VC* Validation Cohort; *EV* Extracellular Vesicles;

## What do all of these biomarker studies tell us?

If miR-21 is a marker of at least 29 diseases, then it cannot be a specific biomarker for any disease. Another way to think about this is as follows: If one did a study with 200 subjects and 100 of them had high miR-21 levels and 100 had low miR-21 levels, what disease does the group with the high miR-21 predict? Lung cancer? Myocardial infarction? Lupus? Because that can’t be known, how can one believe that miR-21 can be a predictive marker for any disease in a general population [76]? The only way miR-21 can be associated with a disease is if the patient’s diagnoses are known *a priori*, as is the construct of biomarker studies. How about miR-21 serving as a biomarker for a prognostic change between low grade and advancing disease? Based on all of the potential reasons that a miR-21 level can be elevated, how much confidence can an elevated miR-21 level in a patient (and elevated relative to what?) be specific to the disease of inquiry versus any other potential health change? Again, there are too many reasons why miR-21 levels may change to be comfortably assigned to the disease of interest.

Another use of miR-21, as seen from the tables, is as one of multiple miRNAs that collectively serve as a biomarker of disease. These sorts of studies tend to find the optimal area under the curve (AUC) based on 2–6 miRNAs [40, 46, 65]. While this approach likely brings more power to the study than a solitary miR-21 approach, it is still difficult to appreciate that miR-21 adds anything to that collection other than to indicate a cell stressor or perhaps a change in the inflammatory cell milieu. As well, miR-21 levels are frequently used in conjunction with other common miRNAs such as miR-155, miR-92a, and miR-122, which also decreases specificity towards a particular disease (Table 1).

One important point about all of these studies is that there is ample evidence that miR-21 expression really is altered in many disease states [11, 20, 77, 78]. That is likely telling us something very fundamental about the expression and function of this highly conserved miRNA. It would seem to suggest that miR-21 is commonly upregulated in a stress environment [1]. Another possibility is macrophages/dendritic cells/circulating monocytes are increased in disease states as part of a global inflammatory response. As the percentage of these cells increase, the levels of miR-21 will increase in tandem in the same bodily fluids. If this is true, then miR-21 may have a very narrow value as a biomarker, where it can be used in conjunction with other cell-specific miRNAs to address the extent of inflammation, if that was a diagnostic factor for a particular disease.

Although we have focused the discussion on miR-21 as a biomarker, all ubiquitous miRNAs should be viewed with caution as potential prognostic biomarkers for any disease [30]. Unfortunately, many miRNAs, such as the let-7 family, miR-10a, miR-22, and others are also both commonly identified as disease biomarkers and common to many different cell types [79]. Each of these three miRNAs are also implicated in a range of neoplastic and non-neoplastic disorders.

## Need to separate the functional importance of a miRNA from its value as a biomarker

miR-21 is clearly involved in key regulatory pathways. Modulating its expression in *in vivo* and *in vitro* studies show clear and important phenotype changes. We may ultimately find that a miR-21 pathway can be successfully targeted for therapeutic intervention. In fact, at least two clinical trials, one in Alport Syndrome (NCT03373786), and another in diabetic wound healing (NCT02581098) are attempting exactly that and may indicate the usefulness in modulating miR-21 levels for efficacy. However, this important role in disease does not make it a useful biomarker to predict these diseases.

## Conclusions

Sadly, miR-21 cannot be considered a specific biomarker for any disease if it is a biomarker of many diseases. While its levels may genuinely vary across bodily fluids in disease states, these variations have no specificity. The miRNA community should stop trying to develop miRNA biomarker studies around miR-21 or other miRNAs with the same characteristics. Future miRNA biomarker researchers should be cognizant of other claims on their miRNA of interest and move toward miRNAs that are more unique.

The best miRNA biomarkers will be those that indicate injury or perturbation to a specific cell type. Already one such miRNA, miR-371a-3p has shown promise as a biomarker for testicular cancer. Whether it will make it into clinical practice is unknown [80, 81]. Other cell specific miRNAs, such as miR-122, a hepatocyte-specific miRNA, is useful in identifying liver injury, while the myomiRs, miR-133, miR-206, miR-208, miR-499 have shown some promise for their ability to identify cardiac injury [30, 82].

In conclusion, miR-21 is a critically important miRNA in health, development, and disease, but based on a significant body of work, is not a useful fluid-based biomarker. Research into this role should not be pursued.

## Data Availability

Not applicable.

## Abbreviations

miRNAs: microRNAs
VMP1: Vacuole Membrane Protein 1
PTEN: Phosphatase and Tensin Homolog
TPM1: Tropomyosin 1
PDCD4: Programmed Cell Death 4
UCSC: University of California, Santa Clara
RPM: Reads per million
PBMCs: Peripheral blood mononuclear cells
DC: Discovery Cohort
VC: Validation Cohort
EV: Extracellular Vesicles
PBMC: Peripheral Blood Mononuclear Cells
CAC: Circulating Angiogenic Cells
AUC: Area under the curve
